# A Novel Pro-Inflammatory Mechanosensing Pathway Orchestrated by the Disintegrin Metalloproteinase ADAM15 in Synovial Fibroblasts

**DOI:** 10.3390/cells10102705

**Published:** 2021-10-09

**Authors:** Tomasz Janczi, Florian Meier, Yuliya Fehrl, Raimund W. Kinne, Beate Böhm, Harald Burkhardt

**Affiliations:** 1 Division of Rheumatology, University Hospital Frankfurt, Goethe University Frankfurt am Main, 60590 Frankfurt am Main, Germany; tomasz.janczi@kgu.de (T.J.); florian.meier@kgu.de (F.M.); yuliya.fehrl@kgu.de (Y.F.); 2 Fraunhofer Institute for Translational Medicine and Pharmacology ITMP, 60590 Frankfurt am Main, Germany; 3 Experimental Rheumatology Unit, Department of Orthopedics, Jena University Hospital, Waldkliniken Eisenberg GmbH, 07607 Eisenberg, Germany; Raimund.W.Kinne@med.uni-jena.de; 4 Fraunhofer Cluster of Excellence Immune-Mediated Diseases CIMD, 60590 Frankfurt am Main, Germany

**Keywords:** mechanotransduction, ADAM15, SIRT1, long non-coding RNA, HOTAIR, TRPV4, pannexin-1

## Abstract

Mechanotransduction is elicited in cells upon the perception of physical forces transmitted via the extracellular matrix in their surroundings and results in signaling events that impact cellular functions. This physiological process is a prerequisite for maintaining the integrity of diarthrodial joints, while excessive loading is a factor promoting the inflammatory mechanisms of joint destruction. Here, we describe a mechanotransduction pathway in synovial fibroblasts (SF) derived from the synovial membrane of inflamed joints. The functionality of this pathway is completely lost in the absence of the disintegrin metalloproteinase ADAM15 strongly upregulated in SF. The mechanosignaling events involve the Ca^2+^-dependent activation of c-Jun-N-terminal kinases, the subsequent downregulation of long noncoding RNA HOTAIR, and upregulation of the metabolic energy sensor sirtuin-1. This afferent loop of the pathway is facilitated by ADAM15 via promoting the cell membrane density of the constitutively cycling mechanosensitive transient receptor potential vanilloid 4 calcium channels. In addition, ADAM15 reinforces the Src-mediated activation of pannexin-1 channels required for the enhanced release of ATP, a mediator of purinergic inflammation, which is increasingly produced upon sirtuin-1 induction.

## 1. Introduction

Chronic inflammation in immune-mediated inflammatory joint diseases is perpetuated by immune cells and tissue-resident fibroblasts in the synovial membrane, which is a specialized connective tissue that lines the inner surface of the capsule of diarthrodial joints [1]. These synovial fibroblasts (SF) develop an aggressive phenotype characterized by an increased apoptosis resistance, a proteolytic attack on extracellular matrix (ECM) components, and infiltrative growth into cartilage and bone as well as the activation pro-inflammatory pathways [2]. Biomechanical loading is an important factor controlling site-specific localization of inflammation and tissue damage, to which activated SF considerably contribute to the inflammatory processes [3,4].

Synovial fibroblasts that are subjected to mechanical loading during the movement of joints perceive transmitted mechanical forces via their ECM receptors, e.g., integrins [5,6]. Focal adhesions, which contain the integrin receptors in their outer layer, provide anchorage to the ECM and transduce mechanical information [7]. Mechanosensitive ion channels also sense mechanical stresses [8]. Thus, the underlying Ca^2+^-ion fluxes play a crucial role in the mechanotransduction pathways, triggering calcium signaling effectors, e.g., the mechanosensitive transient receptor potential vanilloid 4 (TRPV4) [9,10] and calcium/calmodulin-dependent protein kinases (CAMK), known upstream activators of stress-activated c-Jun-N-terminal kinases (JNK) [11,12]. TRPV4 is a Ca^2+^-permeable channel that is involved in the mechanonociception of normal and inflamed joints [13].

The present investigation addresses the elucidation of mechano-induced effects on lncRNA regulation and mechanosignaling pathways in SF with crucial dependency on ADAM15, a disintegrin metalloproteinase with a strongly upregulated expression in the synovial membranes of inflamed joints [14]. ADAM15 is a transmembrane multi-domain protein that binds in vitro to a variety of integrins, e.g., α2β1 and α5β1 [15], and colocalizes with focal adhesion kinase (FAK) at focal contacts in the cell membrane [16]. ADAM15 enhances the cell adhesion of chondrocytes to collagen type II, and its pro-domain-containing fibronectin-type II and III domains bind to native collagen type II [17]. It functions as a trigger of anti-apoptotic signaling pathways, elicited by various death stimuli through the binding and activation of the “survival kinases” Src and FAK [16,18,19].

Emerging evidence shows that lncRNAs are central regulators of inflammatory pathways in RA and osteoarthritis (OA) [20]. They are defined as RNAs of >200 nucleotides in length that are not translated into functional proteins but play key roles in gene regulation [21] and that interact with signaling pathways in human cancers [22]. Our studies identified an ADAM15-dependent downregulation of lncRNA HOTAIR, which is differentially expressed in RA perijoint tissues, e.g., synovial fibroblasts and osteoclasts [23]. One gene targeted by HOTAIR is sirtuin-1 (SIRT1) [24], an NAD+-dependent histone deacetylase, which regulates many physiological functions, including energy metabolism and responses to oxidative stress [25]. Its overexpression in synovial fibroblasts from RA patients contributes to pro-inflammatory cytokine production and apoptosis resistance [26]. Moreover, SIRT1 impacts mitochondrial ATP production [27], and mechanical strain leads to ATP release in various mammalian cells [28]. ATP release occurs through activated pannexin-1 channels (PANX1) that function as an integral component of the P2X/P2Y purinergic signaling pathway and are major contributors to pathophysiological ATP release [29,30].

This study investigated the underlying regulation of lncRNA HOTAIR in mechanosignaling, orchestrated by ADAM15, leading to changes in energy metabolism, oxidative stress response, and purinergic inflammatory pathway activation. The focus on the role of ADAM15 in mechano-induced effector pathways included the elucidation of its crucial impact on calcium signaling by interfering with the mechanosensitive TRPV4 channel, as well as PANX1-mediated ATP release.

## 2. Materials and Methods

### 2.1. Antibodies

Rabbit anti p-Src (Tyr416) #59548S, rabbit anti-pSAPK/JNK (Thr183/Tyr185) #4668S, rabbit anti-p-c-Jun (S63) #91952S, mouse anti-SIRT1 (clone 1F3) #8469S, rabbit anti-p42/44 Erk1/2 #9102S, rabbit anti-phospho-p38 MAPK (Thr180/Tyr182) #9211S and rabbit anti-HDAC1 #2062S antibodies were from Cell Signaling Technology, Frankfurt am Main, Germany. Rabbit anti-ADAM15 ab124698, rabbit anti-β-tubulin AB52901 and mouse anti-GAPDH ab9485 were from Abcam. Goat-anti-ADAM15 AF935 antibody was from R&D Systems; Wiesbaden, Germany. Mouse anti-Ac-lysine antibody (clone 7F8) sc-81623 from Santa Cruz Biotechnology. Rabbit anti-p-pannexin 1 (Y198) ABN1681 was from Merck; Darmstadt, Germany. Rabbit anti-TRPV4 ACC-034 was from Alomone Labs (Jerusalem, Israel).

### 2.2. Cell Culture

Synovial tissue from an arthroscopic synovectomy was obtained during knee or hip joint replacement surgery at the Clinic of Orthopedics, University Hospital Jena, Germany. All patients had given written informed consent and the study was approved by an ethic votum from the Ethics Committee of the Jena University Hospital. All patients met the American College of Rheumatology 1987 criteria for rheumatoid arthritis (RA) (Arnett FC, et al., the American Rheumatism Association 1987 revised criteria for the classification of rheumatoid arthritis (RA). Arthritis Rheum 1988; 31:315–24). The patients (aged between 45 and 71 years) had established RA of >3 years’ duration, with an erosive disease course. The majority of patients (70%) were positive for rheumatoid factor and/or anti-cyclic citrullinated peptide (ACPA). The patients were treated with conventional disease-modifying antirheumatic drugs (DMARDs), such as leflunomide or methotrexate. In accordance with the recommendations of the German Society for Orthopedic Rheumatology, therapy with DMARDs was tapered off prior to joint surgery. Thus, at the time of obtainment of the synovial tissue, none of the patients were receiving antirheumatic treatment, with the exception of prednisolone and/or nonsteroidal anti-inflammatory drugs, at a dosage of ≤10 mg/day. Synovial fibroblasts were isolated and grown in DMEM (Gibco; Thermo Fisher, Schwerte, Germany, #41965-039) supplemented with 10% fetal bovine serum (FBS) (Gibco; #10500-064), 1% L-glutamine (Gibco; #15030-024), 100 units/mL penicillin and 100 μg/mL streptomycin (Gibco; #15140-122) at 37 °C in 5% CO_2_. SF were isolated and characterized, as described in detail earlier [31], and expressed the fibroblast-specific marker proteins cadherin-11 and FAP (fibroblast-activating protein) (Supplementary Figure S1). Fibroblasts between passages 3 and 6 were used for experiments. The chondrocyte cell line T/C28a4 permanently transfected with full-length ADAM15, a deletion mutant without the cytoplasmic domain, or an empty vector were cloned, generated and grown in DMEM/10% FCS, as described in detail previously [17].

### 2.3. Cyclic Biaxial Tensile Strain

For the application of cyclic tensile strain, the Flexcell FX-3000 Tension System (Flexcell International Corp, Hillsborough, USA) was used, which is a computer-based system that uses a vacuum to mechanically strain cells adhering to flexible silicone membranes. A controlled vacuum is applied to a loading station, into which four 6-well culture plates are mounted. SF (3.5 × 10^5^ cells/well) were grown in BioFlex^®^ culture plates coated with type I collagen (Flexcell; BF-3001C) for at least 48 h and were then subjected to continuous mechanical stimulation with an equibiaxial sinusoidal waveform at an elongation of 15% and a frequency of 1 Hz for various time points at 37 °C in 5% CO_2_. Unstimulated cultures were grown under the same conditions but without the straining protocol. Cells were harvested by scraping and used for Western blot and qPCR analysis, as well as NAD+, ROS and ATP assays.

### 2.4. RNAi Silencing in SF

Trypsinized synovial fibroblasts (3.5 × 10^5^ cells/well in a 6-well plate) were treated with 20 nM Silencer Select predesigned and validated small interfering RNAs (Ambion, Thermo Fisher Scientific; Dreieich, Germany) and 20 µL transfection reagent (Synvolux, Leiden, NL; SR-2003-04) in 2.5 mL DMEM, according to the manufacturer’s protocol. The siRNAs used were: for ADAM15, siRNA ID: s16681 (5′GAUCUACUCUGGGAGACAA 3′), SIRT1 siRNA ID: s223591 (5′CAACUACCCAGAACAUA 3′), and HOTAIR siRNA ID: n272229 (5′CAACUCACAGAAUAUAUUU 3′) or the non-silencing siRNA #1. For the double-silencing experiments, cells were first treated with ADAM15 siRNA and, after 8 h, with HOTAIR siRNA. Cells were grown in BioFlex/type I collagen plates for 48 h.

### 2.5. ArrayStar LncRNA Array

SF (3.5 × 10^5^ cells/well) grown in BioFlex/type I collagen plates for 48 h were mechanically strained for 3 h, and total RNA was isolated using the RNeasy kit from Qiagen (#74104). Then, 2 µg of DNase I–treated RNA was reverse-transcribed using the rtStar™ First-Strand cDNA Synthesis Kit from ArrayStar Inc, Rockville, MD, USA (#AS-FS-001). cDNA was amplified in 384-well PCR plates using the nrStar™ Human Functional LncRNA PCR Array from ArrayStar (#AS-NR-004-1), according to the manufacturer’s instructions, in an ABI ViiA™ 7 cycler (Thermo Fisher Scientific). Normalization and subsequent data analysis were performed using software provided by ArrayStar Inc.

### 2.6. Inhibitor Assays

SF (3.5 × 10^5^ cells/well), grown in BioFlex/type I collagen plates for 48 h, were pre-incubated for 30 min with DMEM-containing inhibitors and subjected to mechanical strain for various time points, using SP600125 (50 µM) (S5567-10MG), dasatinib (1 µM) (SML2589-50MG) and GSK2193874 (2.5 µM) (SML0942) from Sigma-Aldrich; Taufkirchen, Germany, KN-93 (50 µM) (#1278)and STO-609 (2.5 µM) (#1551) from Tocris Bioscience; Wiesbaden-Nordenstadt; Germany, TFP (trifluoperazine) (50 µM, Santa Cruz Biotechnology; Heidelberg, Germany, sc-201498), selisistat (50 and 100 µM) (S1541) and carbenoxolone (100 µM) (S4368) from Selleckchem; Munich, Germany.

### 2.7. Semi-Quantitative qPCR

SF (0.35 × 10^6^ cells/well) grown in BioFlex/type I collagen plates were subjected to mechanical strain for various time points. The total RNA was isolated using the RNeasy kit from Qiagen and digested on the column with DNase I (1 µL) for 30 min at 37 °C, according to the manufacturer’s instructions. RNA (500 ng) was reverse-transcribed using M-MLV reverse transcriptase (200 units/1 µL) Promega; M1701) and 1 µL oligo d(T) primers in a total volume of 20 µL for 60 min at 37 °C. cDNA was amplified using SYBR^®^ Green Master Mix (Biorad; Dreich; Germany) and specific primers for HOTAIR sense: 5′ CCTGGCAGAGAAAAGGC 3′, HOTAIR antisense: 5′TACCAGGTCGGTACTGG 3′, SIRT1 sense: 5′ GCAGGTTGCGGGAATCCAA 3′, SIRT1 antisense: 5′ GGCAAGATGCTGTTGCAAA 3′, GAPDH sense: 5′ GAAGGTGAAGGTCGGAGTC 3′, GAPDH antisense: 5′ GAAGGTGAAGGTCGGAGTC 3′, on the DNA Engine Opticon^®^ 2 System PCR-cycler (BioRad). Ct values were normalized to GAPDH, and fold changes were calculated using the 2^−∆∆Ct^ method.

### 2.8. NAD+ Assays

NAD+ was measured using NAD/NADH-Glo detection assay (#G9071) from Promega, according to the manufacturer’s instructions. Briefly, after cell harvesting by scraping and centrifugation in the microcentrifuge 5424 from Eppendorf (Hamburg, Germany), SF (17.5 × 10^3^ cells/50 µL) were lysed with 50 µL of 0.2 M NaOH solution containing 1% dodecyl trimethylammonium bromide (DTAB) (Sigma-Aldrich; #D8639). Then, 50 µL of the lysate was transferred to a new tube and acidified with 25 µL of 0.4-M HCl solution. After incubation for 15 min at 60 °C, resulting in the degradation of NADH, samples were neutralized with neutralization buffer included in the assay (25 µL) and incubated with NAD/NADH-Glo detection reagent (100 µL) in white 96-well plates (Greiner Bio-One; Frickenhausen, Germany) for 30 min at room temperature. The luminescence was measured using the Mithras LB940 plate reader with a 10-second exposure time (Berthold Technologies, Bad Wildbad, Germany).

### 2.9. Reactive Oxygen Species (ROS) Assays

ROS were measured using the ROS-Glo H2O2 Assay (#G8820) from Promega. Briefly, after cyclic straining, cells were scraped together, and 5 × 10^3^ cells (in 80 µL) were transferred to white 96-well plates (Greiner Bio-One) and incubated with 20 µL of a 25 µM H_2_O_2_ substrate solution for 3 h at 37 °C. Then, 100 µL of ROS-Glo detection solution was added and incubated for 30 min, and luminescence was measured.

### 2.10. ATP Assays

ATP was measured using the ATP detection kit from Abcam; Cambridge, UK (ab113849). Briefly: after the stimulation, cells (0.35 × 10^6^) were harvested in 1 mL of PBS, and 100 µL of either supernatant or cell suspension was incubated with a detergent solution (50 μL) in a 96-well white plate for 5 min on an orbital shaker. Substrate solution (50 µL) was added, incubated for 5 min in the dark, and luminescence was immediately measured. A standard curve served as a template for the calculation of ATP concentrations.

### 2.11. Preparation of Cell Lysates and Western Blotting

After stimulation, SF were washed with ice-cold PBS, scraped off and lysed in RIPA buffer (50 mM Tris, pH 7.0, 150 mM NaCl, 5mM EDTA, 1% Triton X-100, 0.25% sodium deoxycholate and complete proteinase inhibitor cocktail (Roche Diagnostics; #11873580001; 40 µL/mL lysis buffer) and phosphatase inhibitor cocktails for inhibiting tyr and ser/thr kinases (Roche Diagnostics; Mannheim, Germany; 10 μL/mL lysis buffer; P5726-1ML and P0044-1ML). Protein concentration was determined using the Pierce BCA protein assay (Thermo Fisher; #23225). Samples (20 µg) were boiled for 2 min at 95 °C and separated by 10% SDS/PAGE (or 14% gels for detection of histones), transferred to a nitrocellulose filter, incubated with primary antibodies (dilution 1:1000) overnight at 4 °C and developed using horseradish peroxidase-conjugated antibodies (dilution 1:2000) (Cell Signaling Technology; anti-mouse #7076S, anti-rabbit #7074S). Signals obtained with chemiluminescence reader Fusion FX (Vilber Lourmat; Eberhardzell, Germany) were evaluated using ImageJ software 1.8.0.

### 2.12. Preparation of Nuclear Fractions

After harvesting by scraping and centrifugation, cells were lysed in 20 mM HEPES, pH 7.2, 150 mM NaCl, 0.5% NP40, containing proteinase (Roche Diagnostics; 40 µL/mL lysis buffer) and phosphatase inhibitor cocktails (Roche Diagnostics; 10 μL/mL lysis buffer) for 20 min on ice with intermittent mixing, and centrifuged at 13,000 rpm for 30 sec at 4 °C. The pellet containing the nuclear fraction was washed twice with the lysis buffer and protein concentration was determined using the BCA protein assay from Pierce/Thermo Fisher.

### 2.13. Co-Immunoprecipitation

SF (4 × 10^6^) or T/C28a4 chondrocyte cells (1.5 × 10^6^), grown in 10-centimeter Petri dishes for 48 h, were washed with ice-cold PBS (Gibco; #14190-094), and lysed in lysis buffer (10 mM HEPES, pH 7.0, 150 mM NaCl), 1% Triton X-100 containing complete proteinase inhibitor cocktail (Roche Diagnostics; 40 µL/mL) and phosphatase inhibitor cocktails (Roche Diagnostics; 10 μL/mL lysis buffer) for 1 h at 4 °C. Lysates were diluted to a concentration of 1 mg/mL and incubated with goat anti-ADAM15 (1 µg/1mg lysate) or rabbit anti-TRPV4 antibodies (1:100 dilution) or mouse IgG as control (1 µg/1mg lysate), and 25 µL Protein G- or Protein A-conjugated agarose bead slurry (Pierce; #20398 and #20333), respectively, under constant agitation overnight at 4 °C. The beads were washed 4 times with lysis buffer, subjected to SDS/PAGE, and subsequent Western blotting was performed as described above.

### 2.14. Enrichment of Plasma Membrane by Cell Surface Biotinylation

Synovial fibroblasts (4 × 10^6^) were grown to subconfluency in Petri dishes (10 cm diameter) for 48 h. Before biotinylation, the medium was aspirated, and the cells were washed twice with PBS. Cells were then incubated with the membrane-impermeable EZ-Link Sulfo-NHS-LC-LC-Biotin from Pierce (0.1 mg/mL in PBS, pH 8.0; #21338) for 15 min at room temperature. Cells were washed again twice with PBS and fixed with 1% paraformaldehyde (PFA) in PBS for 5 min at room temperature, then quenched with 2.5 M glycine for 5 min. After harvesting by scraping and microcentrifugation, cell pellets were lysed in 10 mM HEPES, pH 7.0, 150 mM NaCl, 5 mM EDTA, 1% Triton X-100, containing complete proteinase inhibitor cocktail (Roche Diagnostics; 40 µL/mL) for 20 min on ice and ultrasonicated. Cell debris was removed by centrifugation at 13,000 rpm for 5 min at 4 °C, and the supernatant was transferred to a new tube. Streptavidin-conjugated magnetic beads (10 μL beads/500 μg cell lysate; Thermo Fisher; #88816) and 1U benzonase nuclease (Merck; #70664) were added to the supernatant and incubated under rotation for 2h at room temperature. The magnetic beads were washed thrice with PBS/0.1% Triton X-100. In order to revert PFA-crosslinked protein complexes, the beads were boiled for 45 min at 80 °C in 20 mM Tris/HCl, pH 6.8, 0.5% SDS, 10% Glycerin *v*/*v*, 0,1% bromophenol blue *w*/*v*, and 1% β-mercaptoethanol.

### 2.15. Immunofluorescence

SF (5 × 10^3^ cells) were grown on chamber slides (Falcon BD; Schaffhausen, Switzerland) for 48 h, fixed with 4% PFA in PBS for 5 min and then blocked with PBS, pH 7.2, 1% BSA, 0.1% Triton X-100 for 1 h at room temperature. Cells were incubated with goat anti-ADAM15 (1:50) and rabbit anti-TRPV4 (1:100) antibodies overnight and visualized with Alexa Fluor 488 anti-goat (#A-11055) and Alexa Fluor 594 (#A-21207) anti-mouse conjugated antibodies (1:500; Molecular Probes, Thermo Fisher Scientic) using the Zeiss LSM 80 confocal laser scanning microscope. Nuclei were counterstained with DAPI (4′,6-diamidino-2-phenylindole, dilution: 1:500; Sigma-Aldrich) for 10 min at room temperature. Digital images were processed and adjusted for brightness and contrast using ImageJ. All fluorescence images were acquired under identical conditions.

### 2.16. Statistical Analysis

Statistical significance was determined using Student’s *t*-test when comparing mean values (calculated from triplicate or quadruplicate measurements) from stimulated versus control conditions. The Wilcoxon signed-rank test was used for the comparison of one data set of measured mean values from different individual donors under stimulation, versus the matched data set from the donors under non-stimulated control conditions. *P* values are indicated as follows: * *p* < 0.05; ** *p* < 0.005; *** *p* < 0.0005.

## 3. Results

### 3.1. Downregulation of lncRNA HOTAIR by Mechanical Strain Is Critically Dependent on ADAM15

**Figure 1:**
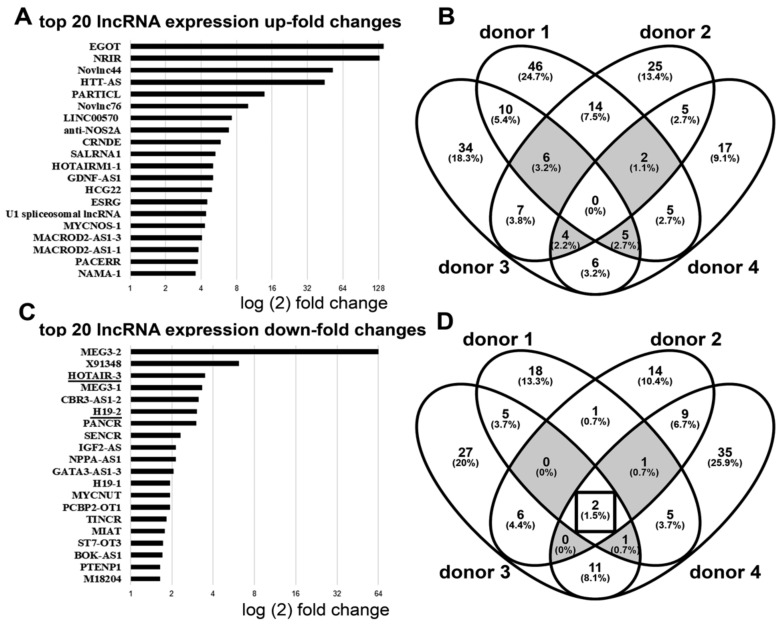
Differential expression of lncRNAs between ADAM15-expressing and non-expressing synovial fibroblasts (SF) under mechanical strain. (**A**–**D**) SF (*n* = 4), either expressing ADAM15 or downregulated with a specific siRNA, were strained for 3 h using the Flexcell System (elongation 15%, frequency 1 Hertz). Reverse-transcribed cDNAs were then amplified in Arraystar lncRNA PCR-plates, and the fold change of up-/down-regulation of gene expression was calculated with the 2^−∆∆Ct^ method. (**A,C**) Top 20 lncRNAs differentially up- and downregulated in ADAM15-expressing versus non-expressing SF. (**B,D**) Venn diagram of all differentially expressed lncRNAs with a 2-fold change up/downregulation, identifying HOTAIR and H19-2 as differentially downregulated lncRNAs in all 4 SF (underlined in **C**; intersection, boxed in **D**). Intersections among 3 donors are shown in grey.

SF from 4 different donors, pretreated with either specific siRNA for ADAM15 or non-silencing control siRNA were strained for 3 h. Subsequently, transcribed RNA was amplified using Arraystar lncRNA qPCR plates coated with primers for 372 disease-relevant lncRNAs, and the overall top 20 up-/downregulated lncRNAs from all 4 donors were determined (Figure 1A,C). Intersections of all differentially expressed lncRNAs (≥2-fold up-regulated) revealed a total of 17 upregulated lncRNAs in synovial fibroblasts from 3 out of 4 of donors, e.g., EGOT, Novlnc76, and MACROD2, but not a single candidate was upregulated in all 4 donors (Figure 1B), indicating some donor-dependent heterogeneity of mechanically upregulated lncRNAs. By contrast, the intersections of all lncRNAs downregulated by ≤2-fold revealed 2 lncRNAs, i.e., H-19 and HOTAIR, in all 4 donors with ~4-fold downregulation (Figure 1D), identifying HOTAIR as a key candidate regulated by mechanical force in the presence of ADAM15.

### 3.2. Strain-Induced SIRT1 Upregulation via ADAM15-Mediated Downregulation of HOTAIR

**Figure 2:**
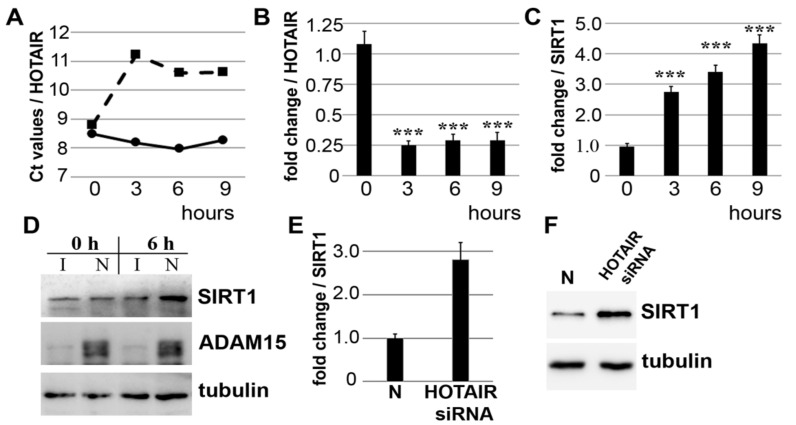
ADAM15-dependent downregulation of HOTAIR under mechanical strain, resulting in the upregulation of SIRT1. (**A**–**D**) SF with prior downregulation of ADAM15 were strained for 0–9 h, and HOTAIR and SIRT mRNA and protein expression was quantified by qPCR and immunoblotting. (**A**) GAPDH-normalized Ct values for HOTAIR from one representative donor, showing higher Ct values, i.e., reduced HOTAIR levels, in ADAM15-expressing SF (dashed line), as compared to SF treated with ADAM15 siRNA (solid line). (**B,C**) Fold change (mean ± SD from 7 donors) of HOTAIR and SIRT1 mRNA in ADAM15-expressing versus non-expressing SF. *** *p* < 0.0005, by Student’s *t*-test, when comparing stimulated versus unstimulated HOTAIR/SIRT levels. (**D**) Immunoblots from SF silenced with ADAM15 siRNA (I) and negative control siRNA (N), showing increased SIRT1 expression in ADAM15-expressing cells after 6 h strain. (**E,F**) Fold change of SIRT1 after HOTAIR downregulation in SF (*n* = 6) when unexposed to mechanical strain by siRNA and negative control siRNA (N), showing increased SIRT1 mRNA (**E**) and protein levels (**F**). Tubulin served as a loading control.

The validation of mechano-induced HOTAIR downregulation was performed by qPCR in SF from 7 different donors. GAPDH-normalized Ct values revealed that HOTAIR was only downregulated in ADAM15-expressing cells, whereas SF with silenced ADAM15 did not show any change in HOTAIR levels after cell straining for up to 9 h, shown in SF from one representative donor (Figure 2A), with a persistent, on average ~4-fold downregulation of HOTAIR in all 7 donors (Figure 2B), clearly showing the requirement of ADAM15 for mechano-induced downstream regulation of HOTAIR.

**Figure A1:**
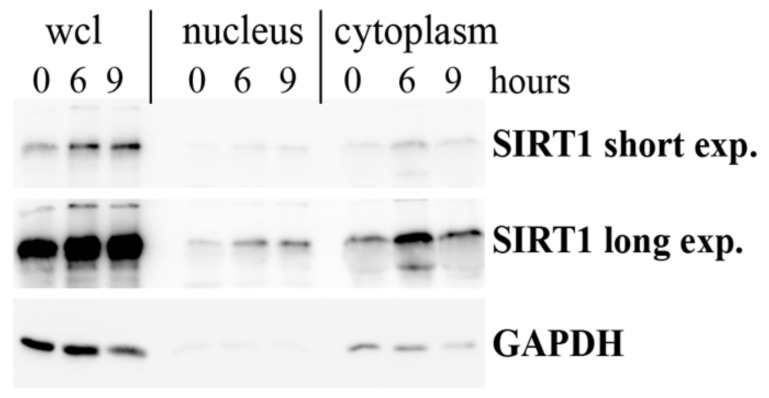
SIRT1 distribution in nucleus and cytoplasm. Immunoblots of whole-cell lysates (wcl), lysates from isolated nuclei (10 µg) and cytoplasm (20 µg) from RASFs, showing increased SIRT1 levels in both nuclear and cytoplasmic fractions after straining for 0–9 h.

Next, we analyzed the mRNA and protein expression of SIRT1, a gene target of HOTAIR, under the abovementioned conditions. The quantification of SIRT1 showed increasingly higher mRNA and protein levels of up to 4-fold in ADAM15-expressing versus non-expressing SF (Figure 2C,D), with increased SIRT1 expression in both nuclear and cytoplasmic fractions (Figure A1). Moreover, HOTAIR silencing of SF unexposed to tensile strain resulted in a ~3-fold increase in SIRT1 mRNA and protein levels (Figure 2E,F), demonstrating that HOTAIR directly affects SIRT1 expression, which is in line with the notion that strain-induced SIRT1 upregulation is directly mediated by the ADAM15-dependent downregulation of HOTAIR.

### 3.3. Impact of ADAM15 and SIRT1 on Histone Acetylation, ROS and NAD+

**Figure 3:**
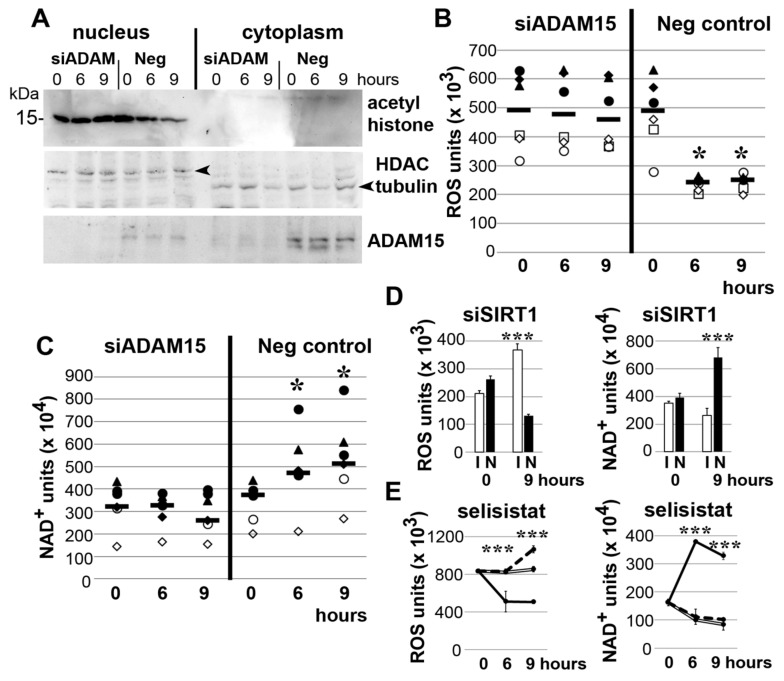
Impact of ADAM15 and SIRT1 on histone acetylation, ROS and NAD+ in mechanically strained SF. (**A–C**) ADAM15 was downregulated by siRNA and a non-silencing siRNA (neg) as control. (**A**) Immunoblots from nuclear and cytoplasmic lysates of SF, showing decreased deacetylated histone after 6 and 9 h strain in ADAM15-expressing cells only. Tubulin and histone deacetylase (HDAC1) served as loading controls. (**B,C**) ROS and NAD+ assays in ADAM15-expressing SF (neg control) as compared to ADAM15-silenced cells. Each symbol represents the mean value of one individual donor, the horizontal bar (-) the median of 6 different donors. * *p* < 0.05, by Wilcoxon signed-rank test for comparison of ADAM15-expressing versus non-expressing SF. (**D**) ROS and NAD+ assays from mechanically strained SF with prior downregulation of SIRT1 by siRNA (I) and a non-silencing siRNA (N) from one representative donor. *** *p* < 0.0005, by Student’s *t*-test for SIRT1-expressing versus non-expressing SF. (**E**) ROS and NAD+ assays from strained SF in the presence of SIRT1 inhibitor selisistat (0 µM; solid line, 50 µM; double line and 100 µM; dashed line) from one representative donor. *** *p* < 0.0005, by Student’s *t*-test, comparing DMEM with the inhibitor. Representative results of at least three independent experiments are shown.

Tensile strain did not induce any change of histone acetylation in nuclear fractions of SF with downregulated ADAM15; however, acetylated histone was reduced by ~3-fold in ADAM15-expressing SF after 6 and 9 h of strain (Figure 3A). Correspondingly, ROS levels were significantly decreased by ~2-fold and, in parallel, NAD+ levels increased by ~2-fold in ADAM15-expressing SF (Figure 3B,C).

As a confirmation that the ADAM15-elicited effects on ROS and NAD+ are mediated by SIRT1, the ROS and NAD+ assays of SIRT1-silenced SF revealed significantly increased ROS and decreased NAD+ levels, compared to SIRT1-expressing SF (Figure 3D). Likewise, the inhibition of SIRT activity by the specific inhibitor selisistat resulted in significantly increased ROS and decreased NAD+ levels (Figure 3E). Together, these data clearly show an impact of ADAM15 on SIRT1-mediated functions in mechanically strained SF.

### 3.4. Impact of JNK on ADAM15-Dependent Mechano-Signaling in HOTAIR/SIRT1 Regulation

**Figure 4:**
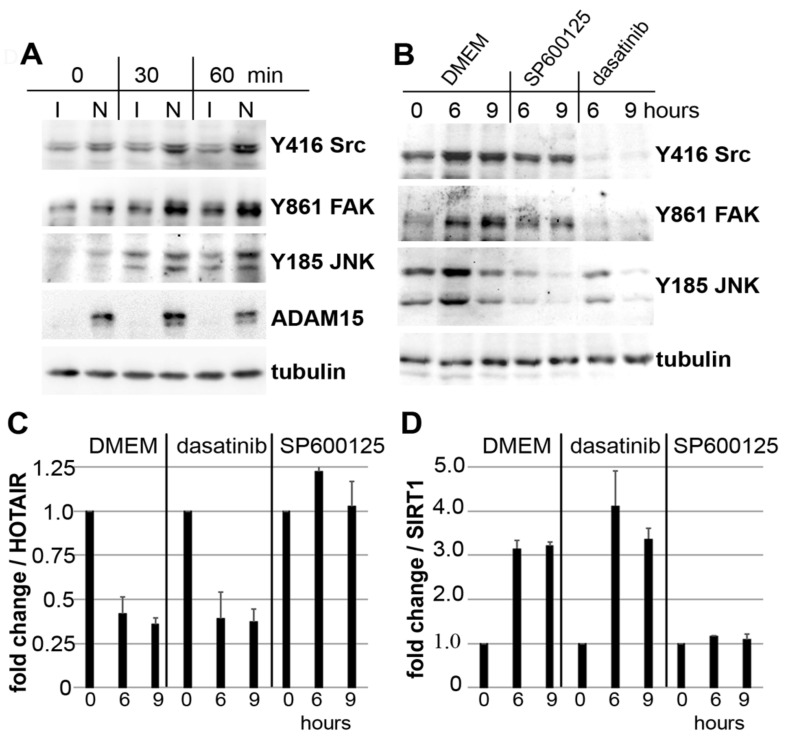
Impact of JNK inhibition on ADAM15-mediated HOTAIR and SIRT1 regulation by mechanosignaling. (**A**) Immunoblots of SF mechanically strained for 30 and 60 min, with prior downregulation of ADAM15 by siRNA (I) and non-silencing siRNA (N) as control, showing ADAM15-dependent activation of Src, FAK and JNK. (**B**) Immunoblots of SF strained in the presence of the JNK inhibitor SP600125 or the Src inhibitor dasatinib. Tubulin served as a loading control. (**C**) Fold change of HOTAIR and (**D**) SIRT1 mRNA levels, calculated by the 2^−∆∆Ct^ method, comparing DMEM control with the respective inhibitor. Mean values ± SD from 6 different donors are shown.

Mechanical strain strongly enhanced phosphorylations of Src at Y416, its target phosphorylation site Y861 FAK, and JNK in ADAM15-expressing SF (Figure 4A). In addition, co-incubation with the Src inhibitor dasatinib or JNK inhibitor SP600125 during 6 and 9 h of strain showed the substantial inhibition of Src/FAK and JNK phosphorylation by their respective inhibitors (Figure 4B).

qPCR analysis revealed that dasatinib does not affect the strain-induced regulation of HOTAIR or SIRT1; however, SP600125 completely abolished the strain-induced downregulation of HOTAIR (Figure 4C), and concomitant upregulation of SIRT1 mRNA levels (Figure 4D), thus revealing the critical role of JNK signaling in ADAM15-dependent HOTAIR/SIRT regulation under mechanical strain.

### 3.5. Mechano-Induced Activation of TRPV4 and CAMK Upstream of JNK

**Figure 5:**
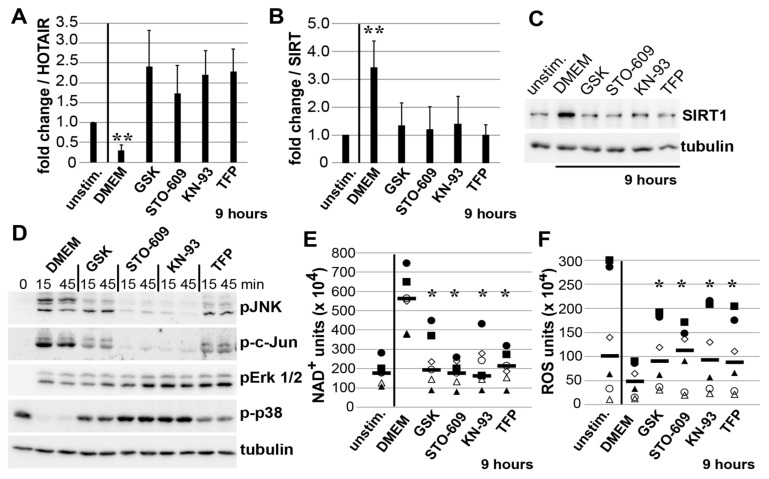
Pharmacological inhibition of TRPV4 and CAMKs inhibits mechano-induced downregulation of HOTAIR, and SIRT1-mediated effects on NAD+ and ROS levels. (**A**) Fold change of HOTAIR and (**B**) SIRT1 mRNA in SF strained for 9 h in DMEM medium or co-incubated with either GSK2193874 (GSK; 2.5 µM), STO-609 (2.5 µM), KN-93 (50 µM) or TFP (50 µM). The fold change of SF from 4 different donors is shown as mean ± SD. ** < *p* 0.005, using Student’s *t*-test, when comparing DMEM versus unstimulated cells. (**C**) SIRT1 immunoblots of strained SF in DMEM and co-incubated with inhibitors. (**D**) Immunoblots from SF strained for 15 and 45 min in DMEM and inhibitors. (**E**) NAD+ and (**F**) ROS assays from SF in DMEM and co-incubated with inhibitors. Each symbol represents the mean value of one individual donor, the horizontal bar (-) the median from 6 different donors. * *p* < 0.05, as determined by Wilcoxon signed-rank test for comparison of inhibitor-treated cells versus DMEM control.

Next, we investigated whether upstream calcium signaling effectors, such as CAMKs, the calcium channel TRPV4, and Ca^2+^-binding calmodulin (CaM) influence the detected, JNK-mediated HOTAIR/SIRT1 regulation. The selective inhibition of TRPV4 by GSK2193874 [32], CAMKK2 by STO-609 [33], CAMKII by KN-93 [34], or calmodulin by TFP [35] all blocked the mechano-induced downregulation of HOTAIR, and even caused its upregulation to various degrees (Figure 5A). Correspondingly, SIRT1 mRNA and protein levels were significantly downregulated by all inhibitors (Figure 5B,C), indicating that HOTAIR/SIRT1 regulation is dependent on the activity of candidate effectors of mechano-induced calcium signaling.

In addition, the specificity of these inhibitors on strain-induced c-jun/JNK phosphorylations revealed inhibition of >95% by STO-609 and KN-93, and ~75% by GSK2193874 and TFP, and no inhibition of the other MAP kinases, ERK1/2 and p38 (Figure 5D).

Correspondingly, the mechano-induced effects on NAD+ levels (upregulated 3-fold) and parallel measured ROS levels (downregulated 2-fold) were completely blocked by all 4 inhibitors (Figure 5E,F), indicating that strain-induced SIRT1 upregulation involves the sequential activation of TRPV4 and CAMKs, finally leading to JNK-mediated HOTAIR downregulation.

### 3.6. Impact of ADAM15 and Calcium Signaling on Strain-Induced ATP Release

**Figure 6:**
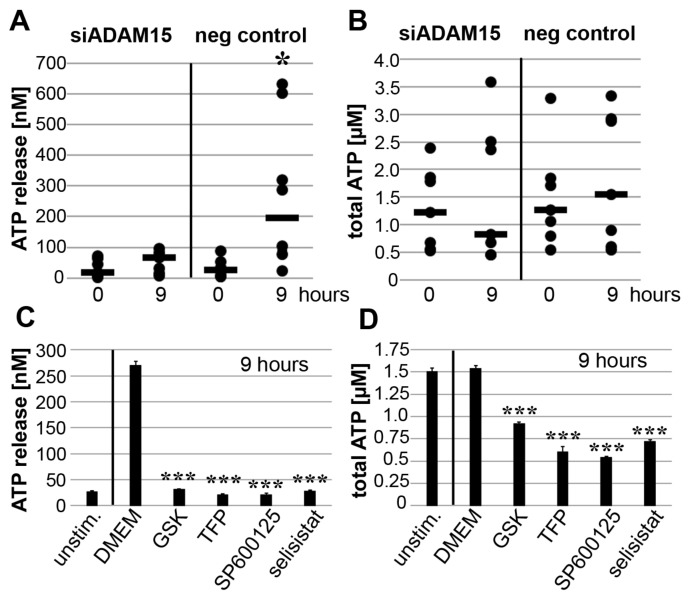
Strain-induced ATP release is dependent on ADAM15 and calcium signaling. (**A**) ATP release and (**B**) total ATP of SF strained for 9 h with prior downregulation of ADAM15 by siRNA and negative siRNA as control. Each dot represents the mean value of one individual donor, the horizontal bar (-) the median of 7 different donors. * *p* < 0.05 by Wilcoxon signed-rank test, comparing ADAM15-expressing versus non-expressing SF. (**C**) ATP release and (**D**) total ATP from SF stimulated with DMEM and inhibitors of TRPV4, CaM, JNK or SIRT1. *** *p* < 0.0005, by Student’s *t*-test, when comparing DMEM with the inhibitor. Representative results out of at least three independent experiments are shown.

Next, we investigated SIRT1-associated effects on mechano-induced ATP production and release. When ADAM15 was expressed, mechanical strain significantly induced ATP release, by ~7 fold from 26.4 nM to 195.6 nM (calculated median from 7 different donors), whereas only minor ATP release was detectable in ADAM15-silenced SF (Figure 6A). In addition, mechanical strain did not influence the total ATP levels in ADAM15-expressing SF but reduced total ATP levels by ~35% in ADAM15-silenced SF (Figure 6B). Likewise, the inhibition of the TRPV4 channel, CaM, JNK or SIRT1 activity by their respective inhibitors completely blocked mechano-induced ATP release, and also inhibited total ATP levels by 40% (Figure 6C,D), indicating the importance of ADAM15 and calcium signaling molecules in mechano-induced ATP release.

**Figure A2:**
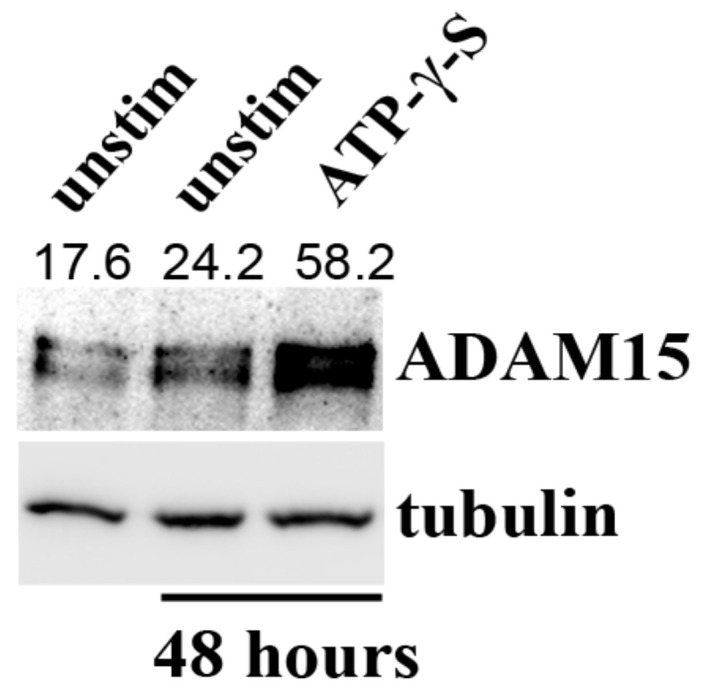
ATP upregulates the expression of ADAM15. Western blot analysis of ADAM15 expression in RASFs stimulated with ATP-γ-S (200 µM) for 48 h. Tubulin served as a loading control. Representative results out of three independent experiments are shown. Densitometric evaluation of protein bands, normalized to tubulin, are shown as numbers above the gel lanes.

In addition to known pro-angiogenic and pro-inflammatory effects, the released ATP may also operate as an autocrine stimulator of ADAM15 expression by SF in a positive feedback loop, showing upregulated signal intensities for the ADAM15 protein band upon 48 h of stimulation with ATP-γ-S (Figure A2).

### 3.7. PANX1 Activity Is Controlled by ADAM15

**Figure 7:**
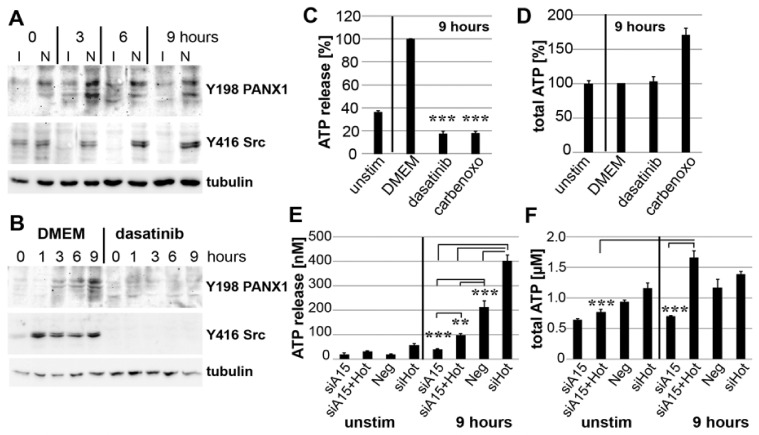
Strain-induced ATP release is dependent on activated pannexin-1 (PANX1). (**A**) Immunoblots from SF, strained for 0–9 h, with prior downregulation of ADAM15 by siRNA (I) or non-silencing siRNA (N), showing increased phosphorylations of PANX1 and Src in ADAM15-expressing SF. (**B**) Immunoblots of strained SF in the presence of dasatinib. (**C**) ATP release and (**D**) total ATP of strained SF in the presence of dasatinib (1 µM) and the PANX1 channel inhibitor carbenoxolone (carbenoxo, 100 µM). Data show the mean ±SD from one representative experiment out of at least 3 independent experiments. *** *p* < 0.0005, by Student’s *t*-test, for comparison of DMEM with inhibitor-treated SF. (**E**) ATP release and (**F**) total ATP from SF with downregulated ADAM15 (siA15), double knockdown of ADAM15/HOTAIR (siA15+Hot), single knockdown of HOTAIR (siHot), and negative siRNA (Neg). ** *p* < 0.005; *** *p* < 0.0005, using Student’s *t*-test.

Next, we investigated whether mechano-induced ATP release involves an ADAM15-dependent activation of the ATP export channel PANX1. SF exhibited markedly enhanced, persistent phosphorylation of PANX1 and Src for up to 9 h strain, compared to ADAM15-silenced SF (Figure 7A). As Src has been shown to activate PANX1 by phosphorylating Y198 [36], SF were strained in the presence of the tyrosine kinase inhibitor dasatinib, which resulted in the complete inhibition of phosphorylations of Src at Y416 and PANX1 at Y198 (Figure 7B). In addition, the inhibition of Src and PANX1 by dasatinib and carbenoxolone [37], respectively, significantly inhibited strain-induced ATP release to the basal level of unstimulated cells, without altering total ATP levels (Figure 7C,D).

To confirm the direct impact of ADAM15 on PANX1-triggered ATP release, both HOTAIR and ADAM15/HOTAIR were silenced by siRNAs. The single knockdown of HOTAIR in ADAM15-expressing strained SF resulted in a significantly increased ATP release. This is likely due to SIRT1-upregulation as a consequence of complete HOTAIR-suppression, as controlled by qPCR (data not shown), which clearly exceeds the ADAM15-mediated regulatory effect imposed by mechanical force alone. However, on the one hand, a double knockdown of ADAM15/HOTAIR resulted in a significant reduction of ATP release to the low levels measured under the conditions of single ADAM15 knockdown (Figure 7E), and, on the other hand, revealed the highest total ATP levels induced by mechanical strain (Figure 7F). Together, our data clearly show both a strain-induced increase in ATP-production via ADAM15/HOTAIR-mediated SIRT1 upregulation, as well as an independent activating impact on the ATP release channel PANX1 by ADAM15, which in the case of its compromised expression leads to an impaired ATP release.

### 3.8. Binding of ADAM15 to TRPV4 Is Critical for Its Membrane Localization

**Figure 8:**
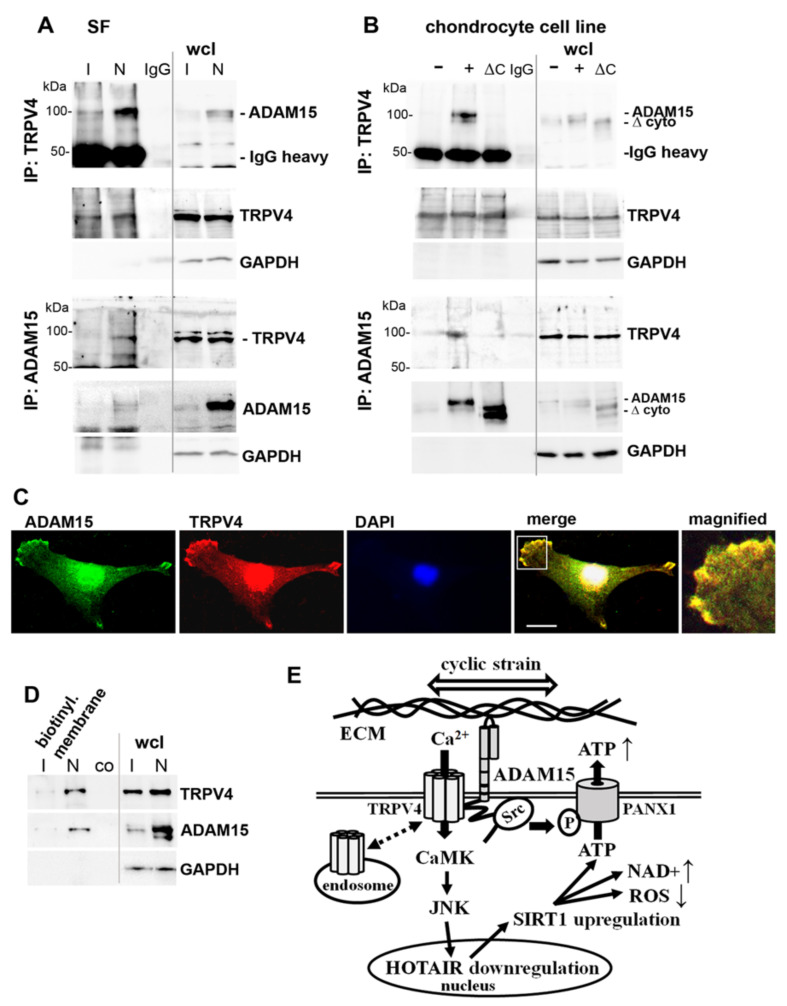
Interaction of ADAM15 with transient receptor potential vanilloid 4 (TRPV4) channel and colocalization in the cell membrane. (**A,B**) Immunoprecipitations (IP) of (**A**) SF with downregulated ADAM15 by siRNA (I) or non-silencing siRNA (N) and (**B**) chondrocyte cell line transfected with full-length ADAM15 (+) or ADAM15 lacking the cytoplasmic domain (∆C), using TRPV4 or ADAM15 antibodies, or IgG as control. GAPDH served as a loading control. wcl—whole-cell lysate. (**C**) Confocal microscopy of double immunofluorescence stainings of SF, using ADAM15 and TRPV4 antibodies. Objective 40x, size bar = 20 μm. The white box marks the magnified area. (**D**) Immunoblots of the cell surface biotinylated and purified membrane fractions of SF with ADAM15-silenced by siRNA (I) or non-silencing siRNA (N), showing TRPV4 in ADAM15-expressing cell membranes only. Co = non-biotinylated cell lysates, purified on streptavidin magnetic beads, served as the background control (N). Representative results out of three independent experiments are shown. (**E**) Diagram of summarized results: cyclic strain results in ADAM15-mediated activation of JNK, the downregulation of HOTAIR and subsequent upregulation of SIRT1, leading to decreased ROS, increased NAD+ levels and ATP release. In parallel, ADAM15-mediated Src activation results in the phosphorylation of PANX1, thereby activating PANX1-mediated ATP release. The interaction with ADAM15 inhibits the constitutive cycling of the mechanosensitive calcium channel TRPV4 to the endosome (dashed double arrow). The knockdown of ADAM15 not only blocks mechanical force-induced JNK- and HOTAIR-dependent upregulation of SIRT1 completely but also eliminates all respective downstream effects on NAD+, ROS and ATP, including its release as a purinergic mediator of inflammation. ECM—extracellular matrix.

Since ADAM15 and TRPV4 are both membrane-integrated molecules, our further studies investigated the hypothesis of their direct interaction. Co-immunoprecipitations (IP) using either ADAM15- or TRPV4-specific antibodies reveal the binding of both proteins in ADAM15-expressing SF (Figure 8A). IPs from T/C28a4 cell lines transfected with full-length ADAM15 (814 amino acids, ~100 kDa) or a deletion mutant lacking the cytoplasmic domain (100 amino acids, ~10 kDa) show that the co-precipitation of TRPV4 with ADAM15 depends on the presence of its cytoplasmic domain (Figure 8B). Accordingly, immunofluorescence stainings of SF demonstrate the colocalization of ADAM15 and TRPV4 in the foci at the cell membrane (Figure 8C). Moreover, TRPV4 detection was confined to enriched cell membrane preparations of ADAM15-expressing SF, while remaining at the detectability threshold in membranes from ADAM15-silenced cells (Figure 8D), indicating that interaction between ADAM15 and TRPV4 is crucial for the cell membrane retention of the calcium channel. A graphic summary of our results is shown in Figure 8E.

## 4. Discussion

In the present study, we identified a strict ADAM15-dependent effect on the biomechanical regulation of the energy sensor SIRT1 and the underlying mechanisms via lncRNA HOTAIR and Ca^2+^-signaling effectors, culminating in the release of ATP in synovial fibroblasts. These cells are key players in tissue invasion and destruction in inflammatory joint diseases through the proteolytic degradation of extracellular matrix proteins in cartilage and bone, eventually leading to the destruction of articular joints [3,38]. Whereas pressure forces in the joint are predominantly perceived via osteocytes in bone and chondrocytes in cartilage [39], the synovial tissue is also exposed to mechanical strain by traction forces in the joint capsule and synovial fluid shear stress during joint movement. To mimic the biomechanical forces in the synovial membrane, we selected an in vitro model using the Flexcell^®^ device for the application of controlled cyclic tensile strain on SF, to study the impact of ADAM15 on induced mechanotransduction.

We identified that ADAM15 is crucial for the mechano-induced downregulation of lncRNA HOTAIR, which then results in an up-regulated expression of SIRT1. Accordingly, HOTAIR has also been described as a mechanosensitive lncRNA, whose repression in stretched aortic valve cells led to an increased expression of calcification genes [40]. The reciprocal upregulation of SIRT1 upon HOTAIR downregulation has been described in studies on the impact of the hepatitis C virus core protein on hepatocyte metabolism [41]. Studies on hepatic insulin resistance also reported an inhibitory effect of upregulated HOTAIR on SIRT1 expression [42]. Thus, the regulatory impact of HOTAIR on SIRT1 seems to be complex and likely context- and/or cell-type-dependent, as illustrated by its potential for SIRT1 upregulation via sponging miRNA34a, which is associated with cardio-protective effects in a murine cardiomyopathy model [43].

In RA synovium, SIRT1 was shown to be upregulated and associated with proinflammatory cytokine production and apoptosis resistance [26]. In this respect, our investigations provide additional new mechanistic insights into mechano-induced SIRT1 upregulation in SF regarding the crucial dependency of ADAM15. Moreover, as ADAM15 is known for various anti-apoptotic effects on synovial fibroblasts [16,18], its newly elucidated impact on mechanically regulated SIRT1 may complement the already revealed spectrum of mechanisms with a modulatory effect on deacetylase activity. The tumor suppressor p53 is a well-studied SIRT1 target [44], whose inactivation by deacetylation causes increased apoptosis resistance to oxidative and genotoxic stress [45,46], thereby likely promoting the aggressive growth of inflamed synovial tissue. Accordingly, the increased invasiveness and cellularity of SF in cartilage and bone erosions has been demonstrated as a consequence of p53 inhibition [47].

A central focus of our investigation was the elucidation of the upstream mechanotransduction pathway; in particular, the molecular interactions with ADAM15. In addition to known ADAM15-mediated Src signaling [16,18,19], the activation of c-jun/JNK, which had already been implicated in the mechanosensing of fibroblasts from other tissues [48,49,50], turned out to be the critical MAPK pathway in the regulation of HOTAIR/SIRT1. Moreover, the described mechanotransduction pathways leading to JNK activation also involve Ca^2+^-dependent mechanisms [50,51]. Thus, studies on mechanosignaling in endothelial cells via direct force application through α1-integrins uncovered stress-induced displacements in the focal adhesion assembly, associated with instantaneous, localized Ca^2+^ influx through TRPV4 channels in the plasma membrane [52,53]. Accordingly, our studies provide unequivocal evidence for the involvement of mechanosensitive TRPV4 channels, linked to the subsequent activation of CAMKs and, finally, to c-Jun/JNK induced in ADAM15-expressing SF by cyclic tensile strain. Thus, the triggering of the β1-integrins via tensile forces in the collagen matrix could be localized to focal adhesions in the cell membrane of endothelial cells [53], a site at which ADAM15 expression, co-localizing with FAK, has been demonstrated [16]. Moreover, the involvement of ADAM15 in Ca^2+^-dependent CaM signaling upon Fas receptor stimulation has been shown in SF [18].

Thus, the revealed colocalization in the cell membrane indicates a potential functional link between TRPV4 and ADAM15 in tensile force perception by SF. Accordingly, our co-immunoprecipitation studies provide conclusive experimental evidence for a direct interaction of the two proteins in critical dependency on the cytoplasmic domain of ADAM15, which is a key factor in promoting TRPV4 enrichment in the cell membrane. The expression of ion channels at the cell surface is essential for their activity and downstream cellular functions, as TRPV4 trafficking to the plasma membrane and its internalization by endocytosis is complex and tightly controlled, involving, e.g., the TRPV4-interacting protein PACSIN 3 [54] or PI3K, PKC, and RhoA signaling pathways [55]. Whereas our studies provide unequivocal evidence for ADAM15-dependent TRPV4 membrane localization, resulting in an upregulated mechano-induced activation of CAMK signaling, elucidation of the precise mechanisms of its impact on TRPV4 membrane-targeting is beyond the scope of the present study and presents an area for future investigation.

Simultaneously with the ADAM15-mediated activation of the mechanosensitive TRPV4, a newly uncovered function in mechanotransduction is its modulation of mechano-induced ATP release through activation of the PANX1 channel by Src. The effector loop of ADAM15-dependent mechanosignaling pathways culminates in the release of ATP as a purinergic mediator, capable of activating a broad spectrum of inflammatory responses (reviewed in [56]). The close proximity of SF to other cells in the synovial tissue, e.g., monocytes/macrophages, dendritic cells, mast cells, and endothelial cells, promotes the pro-inflammatory potential of the released ATP, which is limited by ectonucleotidase activity-dependent metabolization in the extracellular space [56]. However, the effects of ATP are not confined to the stimulation of purinergic receptors involved in inflammasome activation [29] or K_ATP_ channels to induce angiogenesis [57], but instead include the potential for activation of the mannan-binding lectin (MBL) pathway of complement activation by the direct binding of ATP to MBL [58]. The latter aspect is noteworthy as, more recently, mechano-induced complement activation has been described as a mechanism promoting disease chronicity in the experimental mouse model of collagen II antibody-induced arthritis [59].

Moreover, we have shown that ATP-γ-S can upregulate ADAM15 in synovial fibroblasts, thus potentially acting as an autocrine stimulator of ADAM15 expression upon strain-induced ATP release. ADAM15 has also been shown to be upregulated by shear stress via the transcription factor KLF2, thereby promoting the survival of endothelial cells [60]. It is tempting to speculate that the upregulation of ADAM15, triggered by ATP, is a general mechanism that might also occur in other cell types apart from fibroblasts since arterial shear stress could be demonstrated to induce ATP release via the PANX1 channels in human platelets [61]. The positive feedback regulation of ADAM15 expression by ATP is supplemented by the potential of ATP to induce the release of IL-1β [62], a known stimulator of ADAM15 expression [63], via inflammasome activation in neighboring cells. In addition to the release of ATP as a purinergic pro-inflammatory mediator, we also demonstrated an upregulation of the chemokine CCL2 as an earlier-described crucial mediator of mechanoinflammation [3], in mechanically strained SF in strict dependency on ADAM15-regulated SIRT1 (results not shown).

Our elucidation of the critical impact of ADAM15 on the orchestration of mechanoinflammation in SF suggests its potential as a target for therapeutic intervention, which is supported by data on the amelioration of murine collagen-induced arthritis through treatment with ADAM15-specific siRNA [64]. Our investigations reveal the underlying mechanosignaling orchestrated by ADAM15, which exerts cell-adhesive properties to collagen type II [17] and directly interacts with collagen-binding integrins via its disintegrin domain [15], while using cytoplasmic structures to promote the focal cell membrane density of the mechanosensitive TRPV4 channel and the Src-mediated activation of the ATP release channel, PANX1. In this respect, ADAM15 provides a crucial scaffold for mechanosignaling events in synovial fibroblasts. Moreover, its highly upregulated expression in inflammatory diseases, such as rheumatoid arthritis and osteoarthritis, as well as in various cancers [14,63,65], may suggest that this ADAM15-mediated mechanosignaling also occurs in cell types different from (synovial) fibroblasts, contributing to infiltrative growth and the perpetuation of tissue inflammation.

In conclusion, our studies have elucidated a novel and crucial ADAM15-dependent mechano-inflammatory pathway in synovial fibroblasts, which, due to its positive feedback regulation and well-established connection to mechanosensing focal adhesions, may substantially contribute to fueling inflammatory processes.

## Data Availability

The datasets used and/or analyzed during the current study are available from the corresponding author on reasonable request.

## Supplementary Materials

The following are available online at https://www.mdpi.com/article/10.3390/cells10102705/s1, Figure S1: Synovial fibroblasts express FAP (Fibroblast activation protein-α) and cadherin-11.
