# Supplementary material for: A Novel Pro-Inflammatory Mechanosensing Pathway Orchestrated by the Disintegrin Metalloproteinase ADAM15 in Synovial Fibroblasts
Source: Cells. 2021 Oct 9;10(10):2705. doi: 10.3390/cells10102705 (PMC8534551; doi:10.3390/cells10102705)
Supplement: Supplementary file 1 [file cells-10-02705-s001.zip › cells-1395040-supplementary.pdf]

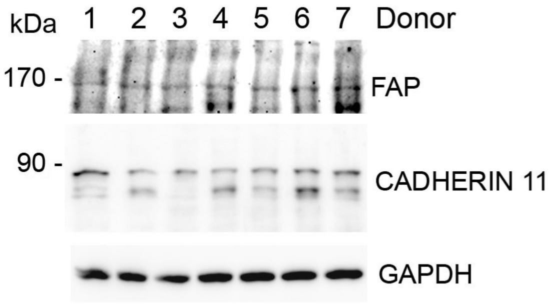

Figure S1. Synovial Fibroblasts express FAP (Fibroblast activation protein-α) and Cadherin-11. Western Blot analysis of FAP and Cadherin-11 expression in synovial fibroblasts from different donors (n = 7). GAPDH was used as loading control.
